# Quality assurance for the NHS abdominal aortic aneurysm screening programme in England

**DOI:** 10.1093/bjsopen/zrab148

**Published:** 2022-02-09

**Authors:** Abigail Campbell, Helena Waggett, Morag Armer, Jo Jacomelli, Jonothan J. Earnshaw

**Affiliations:** 1 Department of Vascular Surgery, Gloucestershire Hospitals NHS Foundation Trust, Gloucestershire, UK; 2 NHS Abdominal Aortic Aneurysm Screening Programme (NAAASP), Public Health England, London, UK

## Abstract

**Introduction:**

The National Health Service Abdominal Aortic Aneurysm Screening Programme (NAAASP) was introduced in England in 2009 to offer ultrasound screening to men over 65 years, in order to reduce aneurysm-related deaths. This study describes the development of a quality assurance (QA) process and conducts an analysis of the first round of QA visit reports. The aim was to identify themes where local providers can target their efforts for improvement.

**Methods:**

Forty-one providers were assessed over 4 years using a process of QA visits adapted from previously established screening programmes. A mixture of qualitative and quantitative methods was used to analyse the 41 QA reports, which identified a range of recommendations for providers. The data were coded for key words and assigned to themes. The number of recommendations per visit report was compared with experience of the providers and performance against national screening standards.

**Results:**

A total of 773 recommendations were made, with an average of 19 per QA visit. Around one third of the recommendations were based on governance and leadership standards, with 43.0 per cent of those based around commissioning and accountability. A significant relationship was seen between number of infrastructure recommendations and performance against standards.

**Conclusion:**

This review of a QA cycle found that sound infrastructure is key to the success of a local provider.

## Teaser

This study describes the development of a quality assurance (QA) process, and conducts an analysis of the first round of QA visits on the National Health Service Abdominal Aortic Aneurysm Screening Programme. A mixture of qualitative and quantitative methods was used to analyse the 41 QA reports. This review of a QA cycle found that sound infrastructure is key to the success of a local provider.

## Introduction

Abdominal aortic aneurysm (AAA) is a leading cause of death in the UK, particularly in men over the age of 65 years^1^. Screening for AAA has been shown to reduce AAA-related deaths, and be cost-effective^2,3^. A National Health Service (NHS) screening programme for AAA was developed, inviting men for a single ultrasound scan to measure the aorta. In England^4^, the Abdominal Aortic Aneurysm Screening Programme (NAAASP) was introduced in April 2009 and fully implemented by April 2013. Since then, every man has been offered screening during the year they turn 65 years old. The aim of the NAAASP is to reduce deaths from AAA among men aged 65 years and over. The design of the programme, and the results of the first 5 years have been published^5^.

All screening programmes aim to identify apparently healthy people who have an increased chance of a disease or condition so that a diagnosis can be made and timely treatment provided^6^. All national screening programmes approved by UK National Screening Committee (UKNSC) must participate in a quality assurance (QA) process^7,8^. The present manuscript describes the design of the QA visit process for NAAASP, and reviews the first cycle of assurance for all AAA screening providers. This study aims to identify what improvements are needed to increase further the quality and safety of AAA screening providers.

## Methods

### Development of quality assurance for abdominal aortic aneurysm

The NAAASP was implemented over 4 years with an incremental roll-out of 41 local providers, each covering a population of about 800 000^4^. In 2013, NAAASP needed a QA model, so a steering group was set up to develop QA for AAA screening. Membership included clinical and QA experts from both the NHS and Public Health England (PHE). The intention was for the QA model to be light touch, but rigorous in the assessment of the quality of AAA screening providers. Models of QA in other well established national screening programmes were considered: antenatal and newborn, breast, bowel, cervical and diabetic eye^9^. The proposed QA model included the development, piloting and evaluation of the AAA QA visit process. Four providers who started screening first participated in the pilot. A multidisciplinary QA team undertook training and conducted four QA visits. A report was produced after each QA visit. Assessment of performance was made against national screening standards, service specification and NAAASP guidance^8,10^. Following evaluation of the process, minor amendments were made, additional professionals trained and the remaining 37 QA visits were completed between 2014 and 2018. The process aimed to be supportive, developmental and help providers maintain and improve on performance.

### Quality assurance review

A mixed-methods approach was undertaken due to the narrative nature of the recommendations and limited data. The 41 QA visit reports were reviewed by two independent assessors not associated with the screening programme (H.W. and A.C.). The date of the QA visit, report author, geographical region and the date the service started were recorded on a proforma. The recommendations were extracted and assigned to section headings and subheadings (*Table 1*). Data were analysed in Microsoft^®^ Excel and R^11^ (Microsoft, Redmond, Washington, USA).

**Table 1:** Subheadings for assessing abdominal aortic aneurysm screening quality assurance reports

Report section	Report subsection
**Governance and leadership**	Commissioning and accountability
	Programme management and co-ordination
	Incident, risk management and escalation
	Audits
	Communication and user feedback
	Oversight and accountability
	Contractual arrangements
	Minimizing harm
	Commissioning and governance
**Infrastructure**	Workforce
	Facilities
	Equipment and IT
	Resilience and business continuity
	Staff
**Identification of cohort**	Population served
	Exclusions
	Identification of cohort
**Invitation, access and uptake**	Informing cohort
	Invitation, access and uptake
	Inequalities
**The screening test – accuracy and quality**	Image QA and feedback
	Internal QA processes and failsafe
	Maximizing the accuracy of the screening test
**Referral**	Vascular network/units
	Failsafe
**Intervention and outcome**	Intervention and outcome audits
	Intervention and treatment
	Diagnosis
	Reporting

IT, information and technology; QA, quality assurance.

QA visit reports include performance against screening standards that measure important parts of the screening pathway, for the most recently available screening year^10^. The first AAA standards were introduced in 2009 and subsequently revised in 2015 and 2016. Each standard has at least one performance threshold assigned. The acceptable threshold is the lowest level of performance that providers are expected to attain. The total number of standards meeting the acceptable threshold were counted for each report.

Descriptive statistics were undertaken for the frequency of recommendations by region, report author, experience of provider (years since implemented) at time of visit, section and subsection^11^. The average number of recommendations per visit was calculated for each variable. The percentage of standards met at the acceptable threshold was plotted against the number of recommendations for the whole report and for each section.

Each recommendation was examined and coded with key words to ensure consistency of language but still allow for different framing of the recommendations. Coded recommendations were then assigned to a primary and secondary theme based on the key words and aim of the recommendation. Similar recommendations were assigned to different themes. The process was repeated three times so that there was consistent extraction of the key words and all emerging themes were recognized. The themes were then reviewed in relation to the report sections to identify if they related to a specific part of the screening pathway or were cross-cutting.

## Results

**Fig. 1:**
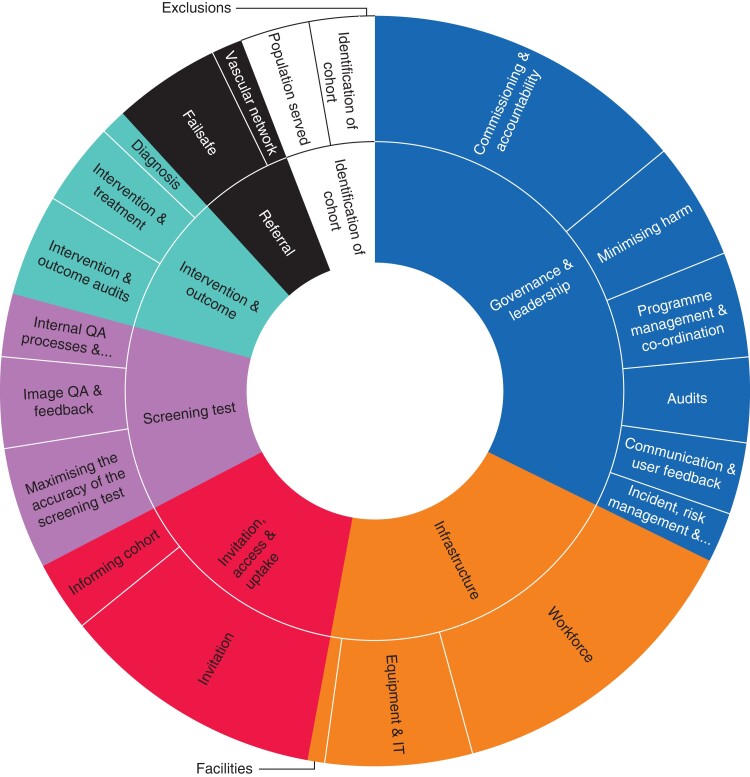
Number of recommendations by report section (inner ring) and subsection (outer ring)

IT, information and technology; QA, quality assurance.

In total there were 773 recommendations made, with an average of 19 per visit (range 4–35) (*Table 2*). The governance and leadership section had the most (251) recommendations, with a further 158 (20.4 per cent) made around infrastructure. Within governance and leadership, 108 (43.0 per cent) of the recommendations concerned commissioning arrangements and accountability, 39 (15.5 per cent) were around minimizing harm and 35 (13.9 per cent) focused on programme management and co-ordination (*Fig. 1*).

**Table 2:** Number of recommendations by report section

Recommendations	Governance and leadership	Infrastructure	Identification of cohort	Invitation, access and uptake	The screening test – accuracy and quality	Referral	Intervention and outcome	Total
**Minimum**	1	1	1	1	1	1	1	4
**Mean**	6	4	2	3	3	2	2	19
**Maximum**	13	9	4	11	7	4	9	35
**Total**	251	158	44	112	92	46	70	773

This included recommendations such as:

Make sure programme standard operating procedures are systematically developed, reviewed and approved in accordance with the Trust quality management system and governance arrangements

Agree with commissioners an annual audit schedule for inclusion in the NHS standard contract and present actions and outcomes to the programme board.

For infrastructure, 99 (65.2 per cent) recommendations focused on the workforce and appropriate staffing time, levels and absence cover. Inadequate resources were frequently noted in clinical skills trainers or internal QA leads (*n* = 18), nurse specialists (*n* = 6) and screening technicians (*n* = 6).

Within the screening invitation section, 88 (78.6 per cent) recommendations concerned access and uptake. This subsection had a wide range of recommendations relating to audit, data reporting, information governance and addressing inequalities, amongst others.

Review the process for accessing translator services and materials.

Analyse uptake data and work with the national screening team to formulate a plan to address areas of need, identifying key stakeholders to support and inform this work

Identification of cohort and referral had the fewest recommendations. For the identification of cohort, 23 (51 per cent) recommendations concerned the population served. These recommendations were about ensuring access to screening for vulnerable or hard-to-reach groups, such as those in prison or with learning disabilities.

Develop a plan to access and encourage registration with a GP for vulnerable/hard to reach groups who would be eligible for screening.

For referral, 36 (78 per cent) recommendations were around failsafe, and strengthening pathways between the provider and medical imaging or the vascular service.

Agree referral pathways with all bordering (commissioned) trusts where patients may choose to be treated.

Intervention and outcome had the third lowest number of recommendations. Of these, 34 (49 per cent) were around intervention and outcome audits.

Establish and implement a clear system around the recording of multidisciplinary team meetings outcomes and how this is fed back to the service.

Develop and implement a systematic audit schedule, across the whole screening pathway, to include review of timelines for referral to clinic and treatment and exception reporting against QA standards.

**Fig. 2:**
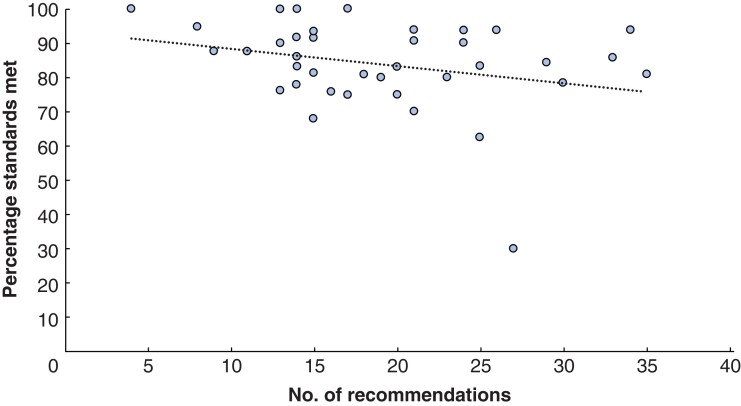
Number of recommendations made against the percentage of standards met at the acceptable threshold

There was a negative correlation between the number of recommendations made and the achievement of the standards at the acceptable threshold, but this was not found to be significant (correlation coefficient –0.279 (95 per cent c.i. –0.54 to 0.03), *P* = 0.769; *Fig. 2*). There was, however, considerable variation. For instance, four providers had a total of 15 recommendations, but the attainment of the standards varied from 68.0–93.5 per cent.

Black dotted line signify the line of best fit.

**Fig. 3:**
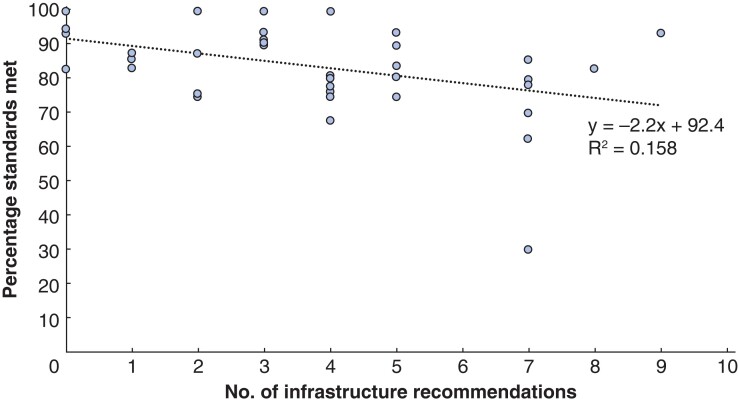
Number of infrastructure recommendations made against the percentage of standards met at the acceptable threshold

Infrastructure was the only report section where there was a significant negative correlation between the attainment of the screening standards and the number of recommendations made (correlation co-efficient –0.397 (95 per cent c.i.–0.63 to 0.10), *P* = 0.010; *Fig. 3*). However, only 15.8 per cent of the variation in the attainment of the standards can be accounted for by the relationship with the number of infrastructure recommendations. Infrastructure recommendations relate to staffing, equipment and facilities. Staffing levels and equipment will have a direct impact on the provider’s ability to deliver screening.

Equation explains how line of best fit calculated. R^2^ is proportion of variation.

**Fig. 4:**
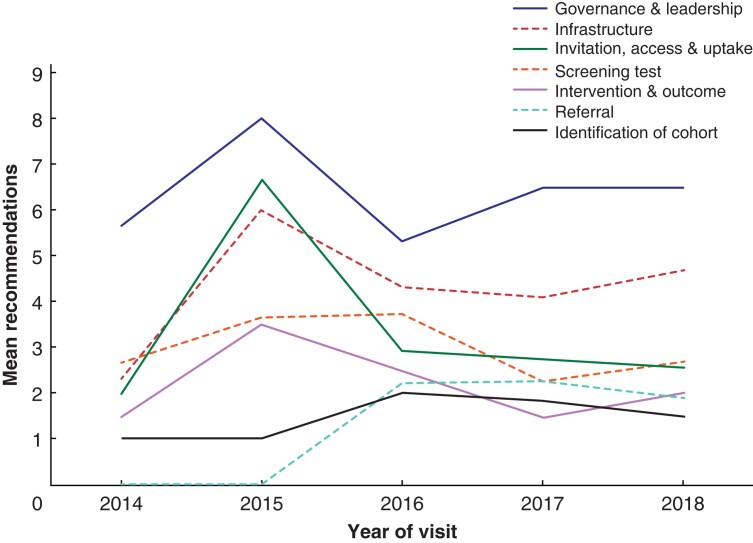
Mean number of recommendations made per visit by report section and year of visit

There was a large increase in the average governance, invitation, infrastructure and intervention and outcome recommendations between 2014 and 2015 (*Fig. 4*). However, the average number of recommendations in each of these sections then dropped in 2016 and has remained constant. There was also an increase in referral recommendations from 2015 to 2016, though the number remained stable over the subsequent 3 years.

**Fig. 5:**
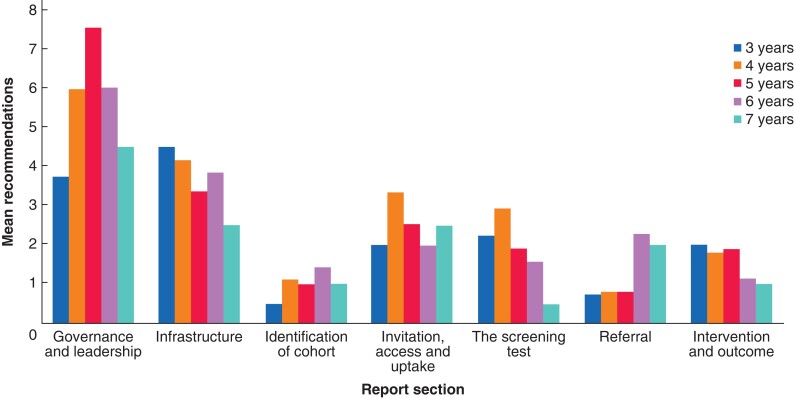
Mean number of recommendations made per visit by report section and age of the screening service at visit

As the experience of the provider may have an influence on the quality of the service, the average number of recommendations by section, and maturity of the provider was calculated (*Fig. 5*). There did not appear to be a relationship between provider experience and the other report sections, but the average number of recommendations was too low to draw conclusions.

**Fig. 6:**
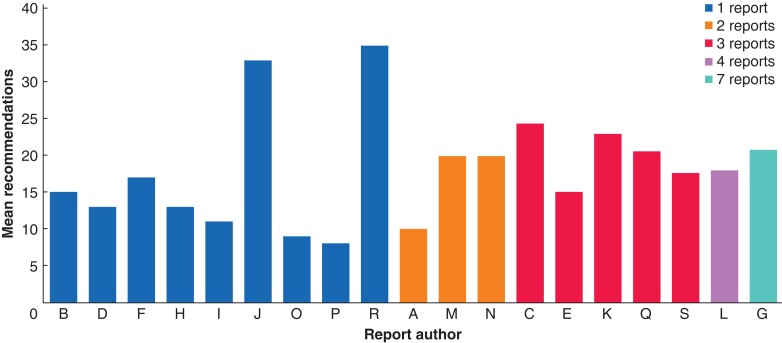
Mean number of recommendations by report author

Reports also varied by their author; the average number of recommendations made by each author was explored. There were 16 individual report authors and three were paired (*Fig. 6*). There was variation in the number of reports written by each author; those writing two or more reports tended to make more recommendations. The average number of recommendations per report per author was 19. However, there were a large number of recommendations by authors J and R. The providers in question were visited in 2015 and were in the first phase of the roll out. This is in contrast to O and P, who visited two of the pilot sites in 2014 and 2015 and made the fewest recommendations. Two providers had been implemented for 4 years, and two for 5 years at the time of the visit. Some of this effect may be that there was no standard report format at that stage and may reflect author style.

**Fig. 7:**
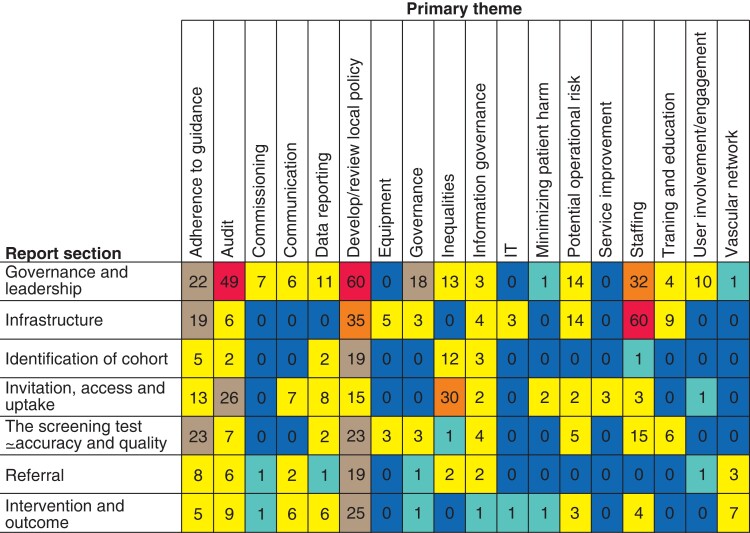
Number of recommendations by primary theme and report section

**Fig. 8:**
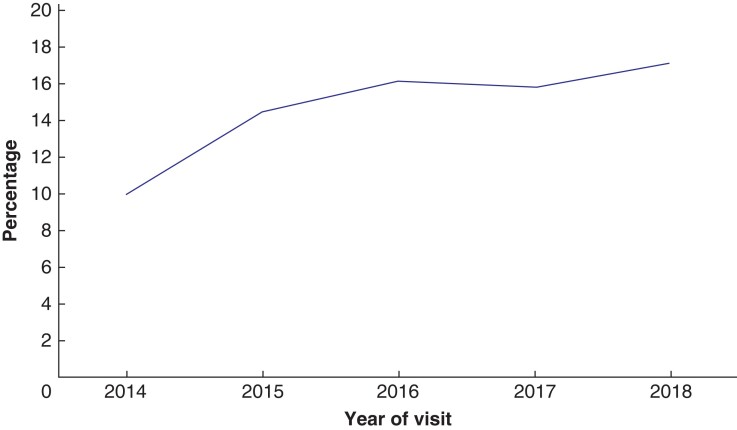
Percentage of recommendations related to inequalities

There were 18 primary themes developed from the key words of the recommendations (*Fig. 7*). When these were mapped against the report sections, four themes were found to be cross-cutting. Adherence to guidance was a primary theme in 95 recommendations, predominantly in governance and leadership, infrastructure and the screening test. Audit was the primary theme in 105 recommendations, with just under half of these in the governance and leadership section, and a quarter around invitation. Developing or reviewing local policy was a principal theme, being mentioned in 195 recommendations. It was a feature of every report section, with the greatest proportion being in governance and leadership. Inequality was an emerging theme which featured strongly and increased between 2014 and 2018 (*Fig. 8*). Inequalities were mentioned most in the invitation, access and uptake section of the reports.

## Discussion

This report describes the creation of a QA process for AAA screening, and review of 41 providers following implementation of a national programme. The principal finding was that almost a third of all recommendations from the QA reports focused on governance and leadership, with 43.0 per cent of these falling under the category of commissioning and accountability. Governance and leadership of a provider is integral to delivery, sustainability and quality. Commissioning and accountability are particularly important, especially in the initial set-up. Interestingly, at the time of the QA visits, the least and most experienced providers had the fewest recommendations relating to governance and leadership. It could be that the more established providers had embedded governance structures which the later providers were able to replicate. Alternatively, it may be that the later providers are yet to experience challenges around governance and leadership due to the early stages of their service. Sharing of good practice identified in QA visit reports could be used to improve governance processes facing younger or challenged programmes. A forum where providers can learn from the experience of others could be valuable for all providers. There was no correlation between performance against national standards and the number of recommendations that were made in the QA visit report. Some services performing well against national standards had a relatively high number of recommendations. This aspect requires further investigation.

An important cross-cutting theme was audit*.* Recent NHS England guidance defines clinical audit as ‘a way to find out if healthcare is being provided in line with standards and lets care providers and patients know where their service is doing well and where there could be improvements’^12^. The guidance indicates that quality-improvement activity should be targeted where it is most likely to improve outcomes for patients. Audit is integral to ensuring the ongoing delivery, quality and effectiveness of any screening programme. All NHS screening programmes are expected to promote audit and learn from the results^12^. NAAASP encourages the use of audit activity to identify challenges, risks and gaps against standards. Services are asked to develop solutions, make changes to practice and re-audit. This present analysis shows an extensive amount of audit activity being undertaken by providers, but not always in a planned way. Development and agreement of audit schedules, including how any service improvements needed would be made, will help providers to identify where the greatest impact can be achieved.

Infrastructure was an important area, particularly around workforce. Some 65.2 per cent of recommendations within this category related to staffing issues, implying that this is inextricably linked with delivering an effective screening programme. The experience of the screening programme was inversely related to the number of recommendations for infrastructure and the screening test. The number of infrastructure recommendations was also inversely related to the percentage of national standards met.

Thematic analysis shows that the most important theme was developing/reviewing local policy, which applied across all report sections, but predominantly in governance and leadership and infrastructure. Concise local policies (which meet national guidance) and clear governance structures are important in the safe and effective delivery of the service. Some providers were not adhering to national guidance and the service specification. Providers need agreed systems and processes in place to develop, ratify, implement and review local policy, guidance and standard operating procedures. Additionally staffing recommendations were also prevalent within these sections, primarily around adequate staffing levels, which affect a provider’s ability to deliver effectively against national standards.

Recommendations mentioning inequalities have increased over time and are frequently related to invitation, access and uptake, but also feature in identification of the cohort and governance. As deprivation levels increase, the less likely men are to attend^13^. This is extremely important in relation to AAA, as men in the most deprived decile are twice as likely to have an aneurysm detected compared with the least deprived decile. PHE released strategies to reduce inequalities in screening programmes in 2018, possibly prompting a rise in the number of inequalities recommendations in subsequent QA visits. Early indications are that this has raised the profile and increased understanding of inequalities. AAAs disproportionately affect men from socioeconomically deprived backgrounds^13^. AAA screening providers therefore have an important role to play in reducing health inequalities. Work to address inequalities in screening is progressing both locally and nationally.

There has been little literature or research on QA of national screening programmes and how this can facilitate and maximize their delivery and effectiveness. This study shows the results of the QA process through a whole screening cycle, and allows other screening programmes the opportunity to compare and evaluate their own processes. The effectiveness of the QA programme can be demonstrated by monitoring further cycles. This study demonstrates how qualitative data can be extracted from QA reports, and make suggestions for improvement: for example, when the QA visit recommendations are implemented, does the provider’s performance against national standards improve?

This project has inevitable weaknesses. First, there was considerable variation in the structure of the QA visit reports before introduction of a standard proforma. The number and grammatical construction of the recommendations in early reports is variable. Limited initial guidance was provided prior to compilation of early reports leading to considerable variation in these. Finally, this analysis concentrated on recommendations, and did not attempt to comment or collate any areas of good practice or themes between screening programmes, which could be an aspiration in the future. The nature of QA analysis tends to lean more towards qualitative rather than quantitative data, which makes comparisons between data sets more complex; this limited the ability to draw quantitative conclusions.

This report details how QA was developed to improve AAA screening services in England, modifying procedures used by other national screening programmes. The study highlights the need for sound governance and staffing of screening programmes, as well as the need to audit procedures for continued development. Providers also need to focus efforts on reduction of health inequalities and strategies to increase uptake. Some of these recommendations may be applicable to other national screening programmes. All these data are freely available within the QA network for shared learning. National networking events and regional AAA screening fora encourage discussion of good practice to improve learning between providers. The more established AAA screening programmes can provide support to later implementers to ensure services learn from each other. The introduction of a standard proforma has improved consistency in QA visit reports. Although this study was not an evaluation of the AAA QA visit process, the wealth of data provided by the visit reports suggests that the AAA QA model should promote quality improvement in local programmes.
